# Bone Marrow Mesenchymal Stem Cells Increase Survival after Ionizing Irradiation Combined with Wound Trauma: Characterization and Therapy

**DOI:** 10.4172/2157-7013.1000190

**Published:** 2014-11-24

**Authors:** Juliann G Kiang, Nikolai V Gorbunov

**Affiliations:** 1 Radiation Combined Injury Program, Armed Forces Radiobiology Research Institute, Bethesda, Maryland, 20889, USA; 2 Department of Radiation Biology, Uniformed Services University of the Health Sciences, Bethesda, MD 20814, USA; 3 Department of Medicine, Uniformed Services University of the Health Sciences, Bethesda, MD 20814, USA

**Keywords:** Radiation, Wound, Stem cell, Bone marrow, Body weight, Wound healing

## Abstract

The aim of this study was to investigate whether treatment with mesenchymal stem cells (MSCs) could improve survival after radiation combined injury. Bone marrow MSCs (BMSCs) were isolated from femurs of B6D2F1/J female mice and were expanded and cultivated in hypoxic conditions (5% O_2_, 10% CO_2_, 85% N_2_) over 30 days. BMSCs were transfused to mice 24 hr after combined injury due to ^60^Co-γ-photon irradiation (9.25 and 9.75 Gy, 0.4 Gy/min, bilateral) followed by skin wounding (CI). Water consumption, body weight, wound healing, and survival tallies were monitored during observation period. Mice subjected to CI experienced a dramatic moribundity over a 30-day observation period. Thus, CI (9.25 Gy)-animal group was characterized by 40% mortality rate while CI (9.75 Gy)-animal group had 100% mortality rate. CI-induced sickness was accompanied by body weight loss, increased water intake, and delayed wound healing. At the 30th day post-injury, bone marrow cell depletion still remained in surviving CI mice. Treatment of CI (9.25 Gy)-animal group with BMSCs led to an increase in 30-day survival rate by 30%, attenuated body weight loss, accelerated wound healing rate, and ameliorated bone-marrow cell depletion. Our novel results are the first to suggest that BMSC therapy is efficacious to sustain animal survival after CI.

## Introduction

Large-scale radiation exposure events in history have shown that irradiated victims often also are subjected to other trauma such as wounds or burns. These combined injuries (CIs) were observed at Hiroshima and Nagasaki, Japan, where 60–70% of victims received thermal burns concurrent with radiation injury [1,2]. At the Chernobyl reactor meltdown, 10% of 237 victims exposed to radiation received thermal burns as well [3]. In animal models of CI including mice [4–15], guinea pigs [16], dogs [17,18], and swine [19], burns, wounds, and infections usually increase mortality after otherwise non-lethal radiation exposures. In rodents, radiation exposure combined with burns, wounds, or infections decreases survival compared to radiation exposure alone [4–14]. Radiation injury (RI) also delays wound closure times [5,7,8,20].

Consequences of CI include accelerated body weight loss, magnified cytokine/chemokine imbalance, systemic bacterial infection, [4], enhanced leukocytopenia, thrombopenia, erythropenia [5,8], acute myelosuppression, immunosuppression, fluid imbalance, macro/microcirculation failure, massive cellular damage [5,20,21], and disruption of vital organ functions. As is the case with radiation exposure alone, these consequences can lead to multi-organ dysfunction (MOD) and multi-organ failure (MOF), the most frequent causes of death after CI [16,22,23]. We hypothesized that (i) intervention on CI-induced delayed wound healing and (ii) enhanced tissue regeneration would improve the survival [21].

Bone marrow mesenchymal stem cells (BMSCs) are bone marrow stroma-derived cells with the capacity to proliferate extensively and to form colonies of fibroblastic cells, namely, colony-forming units-fibroblast, abbreviated CFU-F [24]. Extracellular signaling factors including growth factors and cytokines promote and/or maintain BMSC self-renewal due to their genes of *oct-4, sox-2, and rex-1* [25]. It was reported that BMSCs promoted wound healing when these cells were injected to the wounded area [26]. SOD gene-transfected BMSCs improved survival after RI [27] while Hu et al. [28] reported that 3 doses of BMSCs improved mouse survival after RI. It is documented that bone marrow transplantation restored the intestinal mucosal barrier after RI [29] and survival after CI [9]. Moreover, autologous mesenchymal stem cells derived from adipocytes [30] or bone marrow [31] improved wound repair after severe radiation burn on skin. Therefore, it was of interest to investigate whether treatment with BMSCs increased survival in our model of radiation combined with skin-wound.

In this communication, mice were given Co-60 *γ*-irradiation followed by skin-wounding. They were then treated with BMSCs. This therapy significantly improved mouse survival, accelerated skin-wound healing, and recovered bone marrow after CI, suggesting that BMSC therapy is a tangible remedy to substantiate target-based therapy for the complex molecular responses to CI.

This report, which is intended to stimulate interest in advancing research on BMSCs in support of approval for treatment of CI-induced bone marrow injury by the U.S. Food and Drug Administration, provides data from an experimental CI animal model designed to demonstrate the efficacy of BMSCs as an effective countermeasure against complicated forms of radiation-related injuries, when recuperation requires substantial tissue regeneration.

## Materials and Methods

### Ethics Statements

Research was conducted in a facility accredited by the Association for Assessment and Accreditation of Laboratory Animal Care International (AAALACI). All procedures involving animals were reviewed and approved by the AFRRI Institutional Animal Care and Use Committee. Euthanasia was carried out in accordance with the recommendations and guidance of the American Veterinary Medical Association [32,33].

### Animals

B6D2F1/J female mice (The Jackson Laboratory, Bar Harbor, ME) were maintained in plastic microisolator cages on hardwood chip bedding. Commercial rodent chow and acidified tap water were provided ad libitum at 12 to 20 weeks of age. Animal holding rooms were maintained at 21°C ± 1°C with 50% ± 10% relative humidity using at least 10 changes/h of 100% conditioned fresh air. A 12-h 0600 (light) to 1800 (dark) full-spectrum lighting cycle was used.

### Gamma irradiation

Mice were given 9.25 and 9.75 Gy [5,20] whole-body bilateral ^60^Co gamma-photon radiation, delivered at a dose rate of 0.4 Gy/min, while held in vertically stacked, ventilated, four-compartment, acrylic plastic boxes that provided electron equilibrium during irradiation. Empty compartments within the boxes were filled with 3 inch-long, 1 inch-diameter acrylic phantoms to ensure uniform electron scattering. The mapping of the radiation field was performed with alanine/EPR dosimetry [34] using standard alanine calibration sets from NIST and National Physical Laboratory of UK. The mapping provided dose rates to water in the core of the acrylic phantom (3 inches long, 1 inch in diameter) in each compartment of the mouse rack on the day of the mapping. The field was uniform within ± 1.8% over all of the 120 compartments. The exposure time for each irradiation was determined from the mapping data; corrections for the ^60^Co decay and the small difference in the mass energy absorption coefficients for water and soft tissue were applied. The accuracy of the actual dose delivery was verified with an ionization chamber adjacent to the mouse rack, which had been calibrated in terms of dose to soft tissue in the cores of mice.

### Wound

Skin surface injuries were performed on the shaved dorsal surface of mice. Animals receiving skin wounds were anesthetized by methoxyflurane inhalation. Within 1 h after irradiation, mice were anesthetized by methoxyflurane prior to wounding. A non-lethal 15% total-body surface area (TBSA) wound was administered approximately 20 mm from the occipital bone and between the scapulae using a stainless steel punch on a Teflon-covered board cleaned with 70% alcohol before each use. The panniculuscarnosus muscle and overlying skin (approximately 24 mm in length and about 15 mm in width) were removed [4].

### Bone marrow stem cells

Bone marrow stem cells were obtained from 3- to 4-month-old B6D2F1/J female mice using a protocol adapted from STEMCELL Technologies, Inc., and were expanded and cultivated in hypoxic conditions (5% O_2_, 10% CO_2_, 85% N_2_) for approximately 30 days in MESENCULT medium (STEMCELL Technologies, Inc.) in the presence of antibiotics. Phenotype, proliferative activity, and colony-forming ability of the cells were analyzed by flow cytometry and immunofluorescence imaging using positive markers for mesenchymal stem cells.

### Treatment with bone marrow stem cells

Bone marrow stromal cells (BMSCs) in a volume of 0.4 ml Dulbecco’s Modified Eagle’s Medium (DMEM) were injected i.v. to mice 24 hr after CI. The vehicle given to control mice was sterile DMEM.

### Survival

Survival studies were performed with 20 mice per group. Gross appearance, general health, and survival of each mouse were followed by visual inspection 3–4 times daily for 30 d [5].

### Water consumption

Drinking water was provided in a steam-sterilized graduated bottle and placed on the top of each cage, in which 4 mice were housed. The drinking bottle was connected to a sipping tube with a metal ball inside to prevent water leakage. The volume of water was measured daily for the first 7 d. The average volume drunk per mouse per day was calculated [5].

### Body-weight and wound-closure measurements

The body weight of each mouse was measured at the start of the experiments and at the time of wound-closure assessments on days 1, 3, 7, 14, 21 and 28 after skin wounding. Wound-size measurements were made with calipers. The average area of each wound was calculated as area= π × diameter A/2 × diameter B/2 (A and B represent diameters at right angles to each other) [5].

### Histology

Bone marrowpecimens were immediately fixed in 10% phosphate-buffered formalin upon removal, and then embedded in paraffin, sectioned transversely and stained with hematoxylin and eosin (H&E). Tissue imaging and cell counts were performed with the NanoZoomer 2.0 from Hamamatsu Photonics K.K. (Hamamatsu, Japan) [4].

### Phenotype Control of BMSCs

Phenotyping of the cultured cells was conducted using immunofluorescence labeling of cell surface proteins with antibodies against conventional positive markers of BMSCs (e.g., Sca-1, Stro-1, CD44) and negative markers (e.g., CD34). That was followed by either flow cytometric or fluorescence microscopy analyses

For flow cytometric analysis, the harvested cells (3 specimens per group) were re-suspended in Hank’s Balanced Salt Solution (HBSS) containing 10% Fetal Bovine Serum (FBS) using polypropylene tubes. Then the cells were incubated either with rat anti-mouse CD44-biotin IgG (BioLegend; San Diego, CA), rat anti-mouse Ly-6A/E (Sca-1)-biotin IgG (BioLegend; San Diego, CA), or mouse IgG anti- CD34 conjugated with Alexa Fluor^®^ 647 (eBioscience; San Diego, CA) for 20 min at room temperature. Isotype controls were Mouse IgG conjugated with Alexa Fluor^®^ 647 (BioLegend San Diego, CA) and rat IgG-biotin (BioLegend San Diego, CA). Biotin counter-conjugates were streptavidin-Pacific Blue^™^ conjugate and streptavidin-Alexa Fluor^®^ 647 conjugate (Life Technologies Inc., CA). The cells were analyzed with the BDTMLSRII Flow Cytometer (BD Biosciences Co., www.bdbiosciences.com). The histogram data represent cell counts per 10,000 events. Positive marker-specific immunofluorescence of BMSCs was distinguished from the isotype immunofluorescence (negative control for nonspecific immunoadherence) in all samples.

For immunofluorescence confocal imaging, BMSCs (5 specimens per group) were grown on chambered coverglass (Thermo Fisher Scientific, Inc.). Then they were fixed in 2% paraformaldehyde, processed for immunofluorescence analysis and analyzed with fluorescence confocal microscopy. Normal donkey serum and antibody were diluted in phosphate-buffered saline (PBS) containing 0.5% BSA and 0.15% glycine. Any nonspecific binding was blocked by incubating the samples with purified normal donkey serum (Santa Cruz Biotechnology, Inc., Santa Cruz, CA) diluted 1:20. Primary antibodies were rat anti-mouse CD44-biotin IgG (BioLegend; San Diego, CA), rat anti-mouse Ly-6A/E (Sca-1)-biotin IgG (BioLegend; San Diego, CA), mouse anti-Stro1 IgM (Santa Cruz Biotechnology Inc.), and goat IgG against glycerol-3-phosphate dehydrogenase 2 (GPD2) (Santa Cruz Biotechnology Inc.). Secondary antibodies were anti-IgM mouse goat conjugated with Alexa Fluor^®^488 and donkey anti-IgG goat conjugated with Alexa Fluor^®^594 (Life Technologies Inc., CA). Biotin counter-conjugates were streptavidin-Pacific Blue^™^ conjugate (Life Technologies Inc., CA). For nuclei counterstaining we used Heochst 33342 (Molecular Probes, Inc., Eugene OR) that was diluted to 1:3000. Negative controls for nonspecific binding included normal goat serum without primary antibody or with secondary antibody alone. Five confocal fluorescence images (per specimen) were captured with a Zeiss LSM 710 microscope. The immunofluorescence image analysis was conducted as described previously [35].

### Statistical analysis

Parametric data are expressed as the mean ± s.e.m. For each survival experiment, 10–11 mice per group with one repeat were tested with vehicle or BMSCs. Survival analyses were performed using the log-rank test. For cell analysis, one-way ANOVA, two-way ANOVA, Bonferroni inequality test, and Student’s t-test were used for comparison of groups, with 5% as a significant level.

## Results

### Characterization of BMSCs

**Figure 1:**
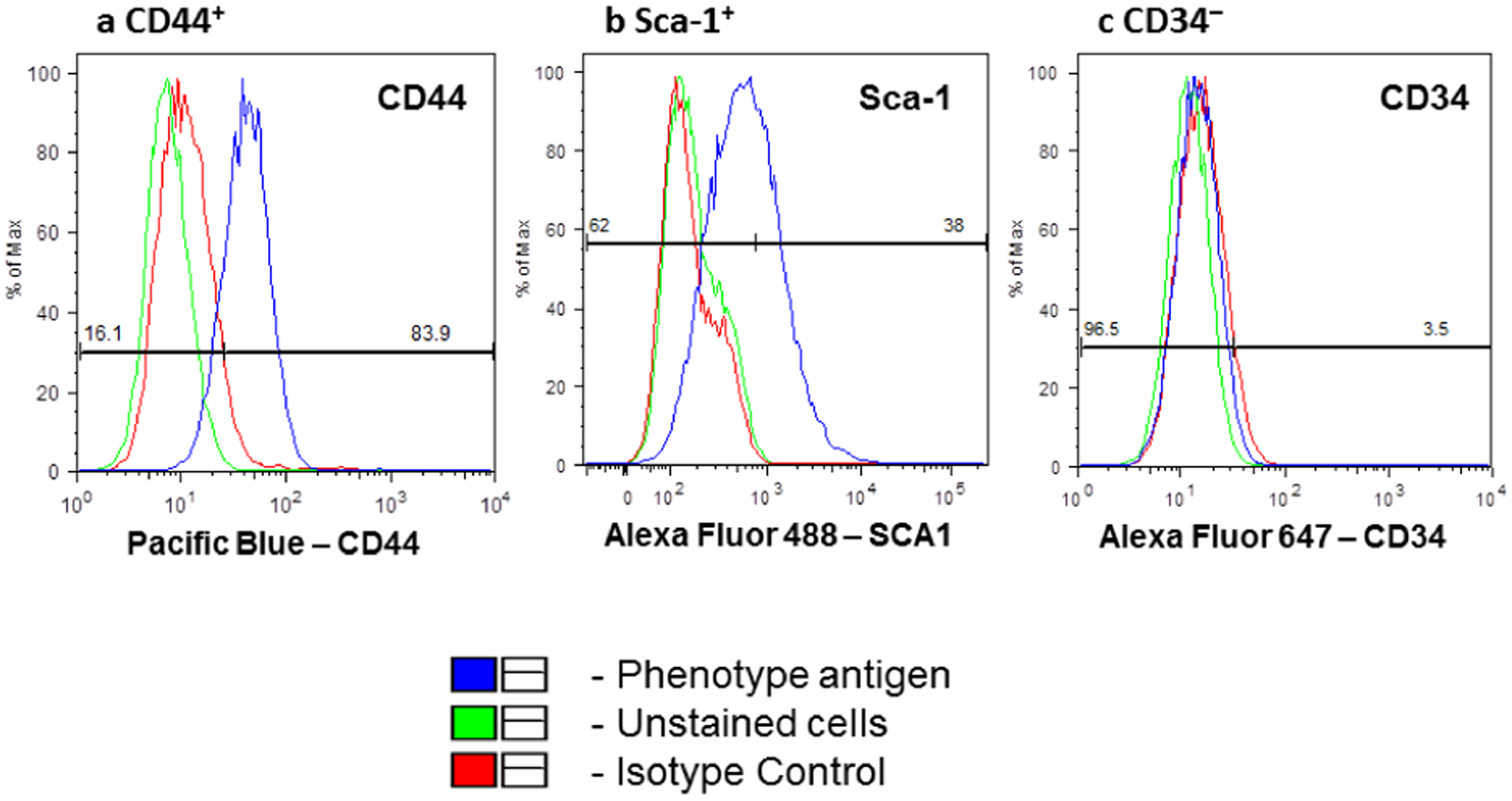
Flow Cytometry Phenotype Analysis of mouse BMSCs - CD44+, Sca-1+, and CD34− Immunofluorescence analysis of the expression of BMSC surface markers for phenotype cell assessment. BMSCs were obtained from bone marrow. They were cultivated for 45 days and subjected to 4 passages. (a-c) BMSCs expressed CD44 and Sca-1 but not CD34

BMSCs express CD44 and Sca-1 but not CD34 [27]. To validate BMSCs, these cells were stained with antibodies direct to CD44, Sca-1, and CD34. The flow cytometric analyses showed the presence of CD44 and Sca-1 while CD34 was absent (Figure 1a–c). To further confirm their identity, cells with immunofluorescent staining were conducted.

**Figure 2:**
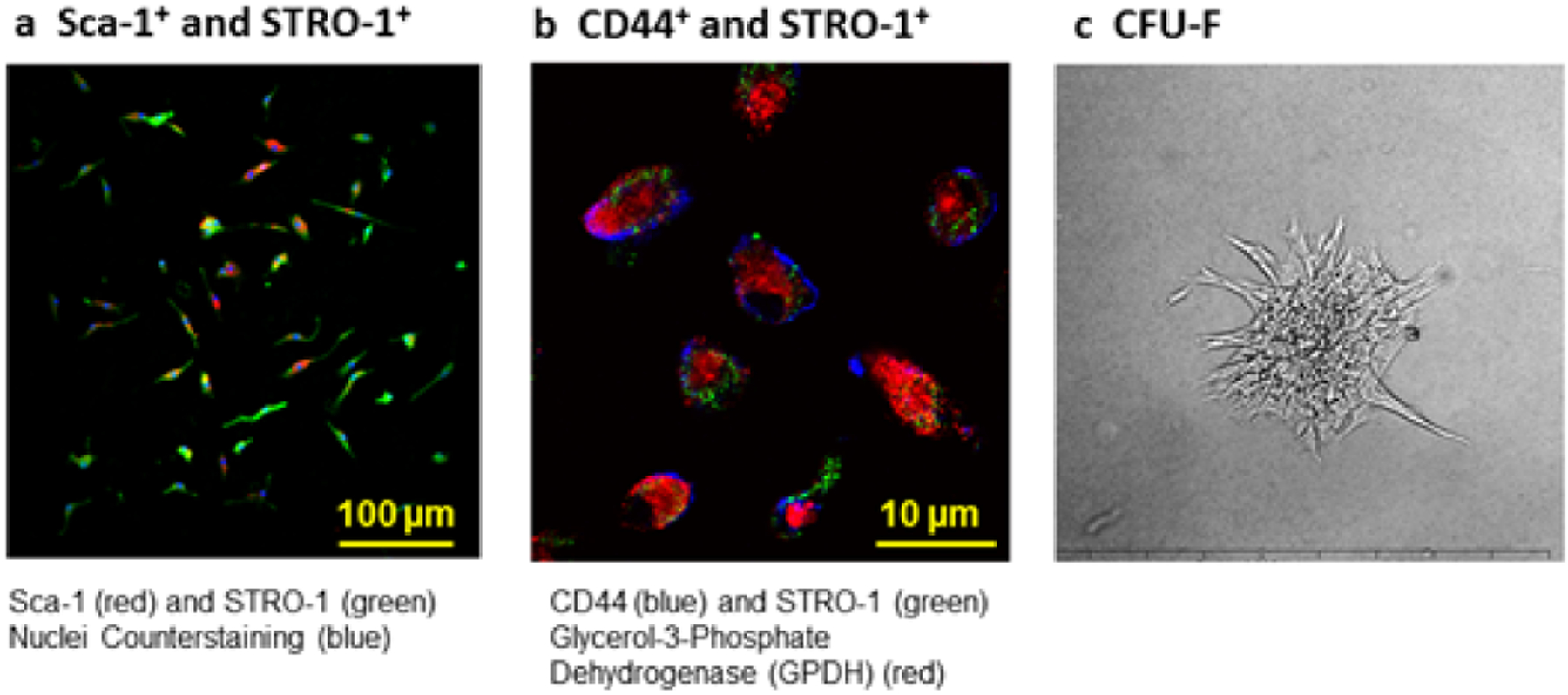
Flow cytometric analysis and colony-forming unit of BMSCs. (A) Projections of Stro-1 (green) and Sca-1 (red). Counterstaining of nuclei is in blue (Heochst 33342). (B) Projections of Stro-1 (green), CD44 (blue), and mitochondrial GPD2 (red). (C) A representative colony of BMSCs.

As shown in Figure 2a and 2b, cell phenotype analysis with Stro-1+ further indicated that the cells were bone marrow stromal cells (BMStroCs). BMSCs form colony-forming unit fibroblasts [24]. Figure 2c depicts a representative colony, confirming their characteristics of BMSCs. It took 28.7 ± 6.3 hr to double BMSC numbers.

### Survival

**Figure 3:**
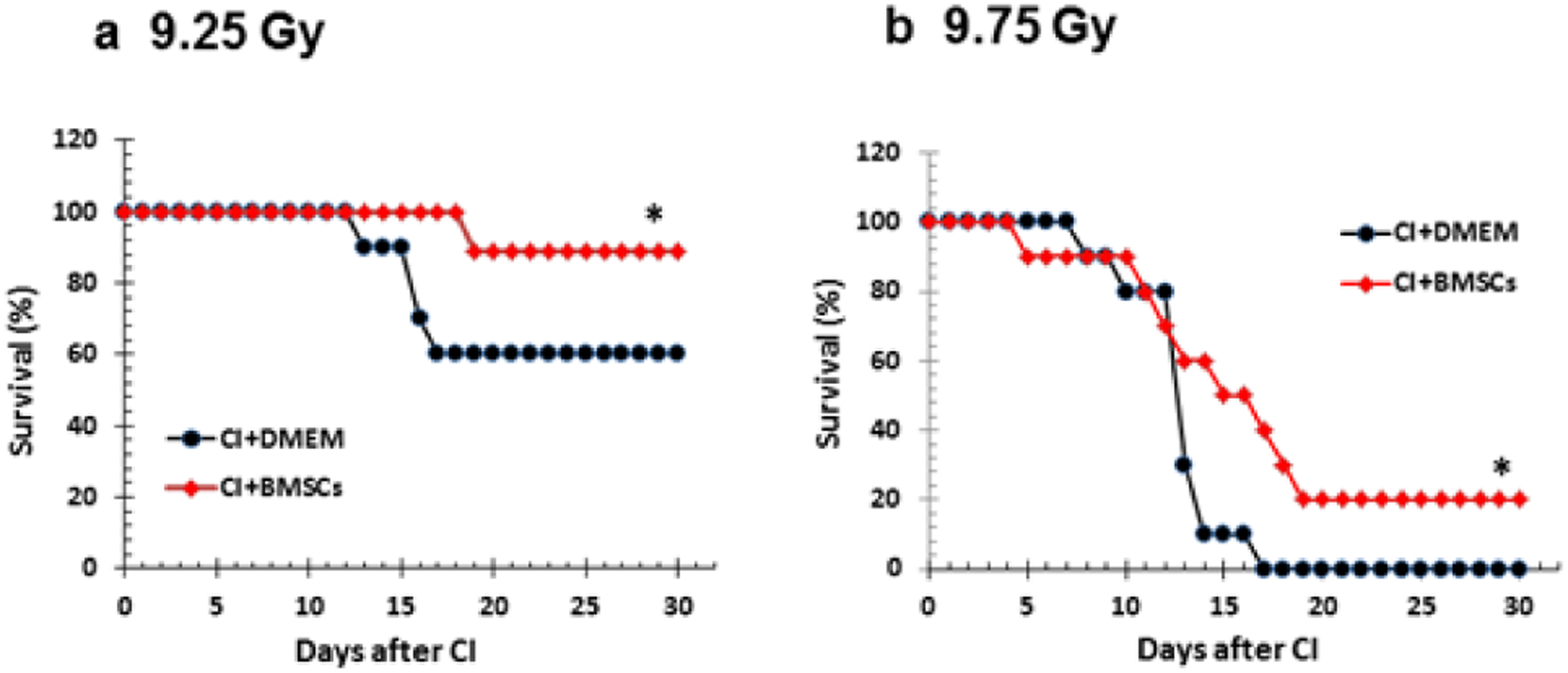
BMSCs improved survival after whole-body ionizing irradiation combined with skin wound (CI). B6D2F1/J mice were exposed to *γ*-radiation followed by 15% TBSA skin-wound trauma. BMSCs (approximately 3 × 106 cells per mouse) were injected via tail vein 24 h after CI. N=10–11 per group and repeated once. (A) 9.25 Gy. 30% survival improvement was observed. *P<0.05 vs. CI +DMEM. (B) 9.75 Gy. 20% survival improvement was observed. *P<0.05 vs. CI+DMEM. CI: Radiation + Wound; DMEM: Dulbecco’s Modified Eagle’s Medium

It was reported that BMSCs promoted wound repair when these cells were injected to the wounded area [26]. Therefore, BMSCs were tested in our CI animal model of radiation combined with skin wound. Mice with skin wound (15% total-body-surface area) alone display 100% survival over a 30-day observation period [7]. In CI mice, treatment with BMSCs 24 h after CI increased survival from 60% to 90% (p<0.05) as shown in Figure 3a.

Similar results were observed when mice were exposed to 9.75 Gy. Skin wound following irradiation at 9.75 Gy displayed 0% survival. In CI mice, treatment with BMSCs 24 h after CI, however, enhanced 30-day survival from 0% to 20% (p<0.05) as shown in Figure 3b. The average survival time (ST_50_) was increased by 4 days (vehicle: 11 days vs. BMSCs: 15 days, P<0.05), suggesting presence of therapeutic effects of BMSCs. We noticed that surviving CI mice treated with BMSCs did not develop tumors. They looked as well as those surviving CI mice treated with vehicle.

### Body weight

**Figure 4:**
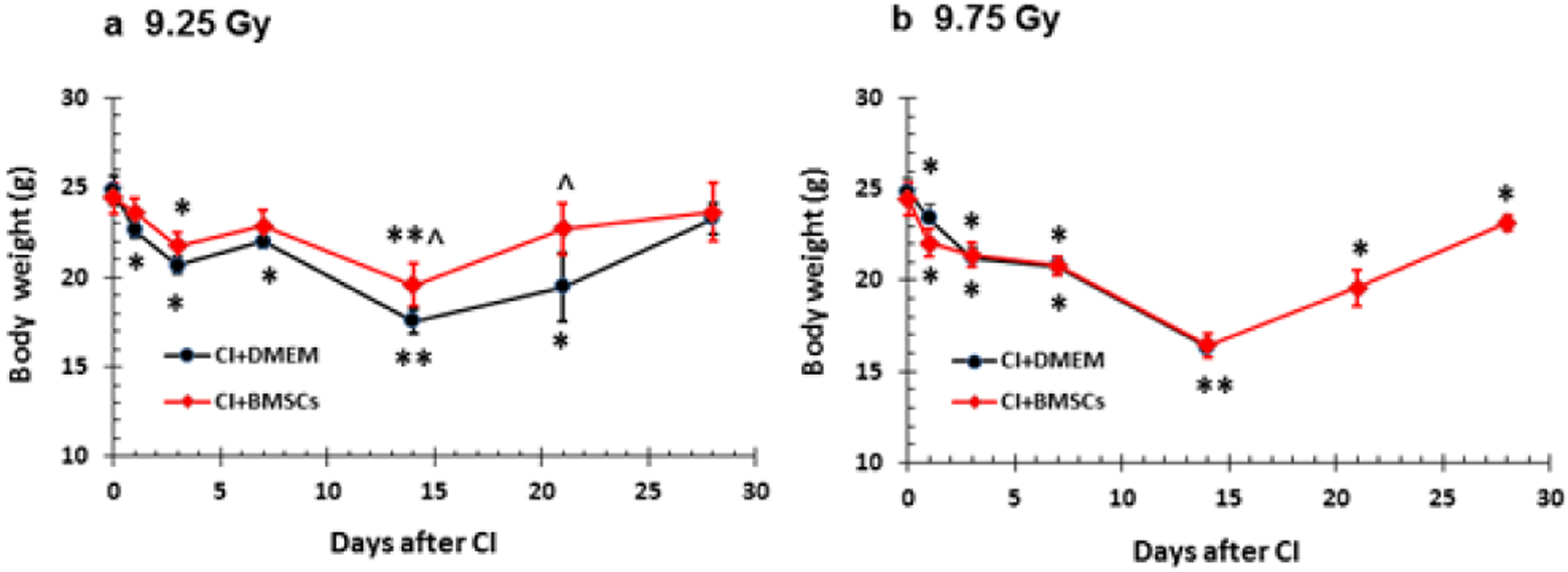
BMSCs recovered body weights after whole-body ionizing irradiation combined with skin wound (CI). B6D2F1/J mice were exposed to *γ*-radiation followed by 15% TBSA skin-wound trauma. BMSCs (approximately 3 × 106 cells per mouse) were injected via tail vein 24 h after CI. N=10–11 per group and repeated once. a. 9.25 Gy. b. 9.75 Gy. *P<0.05 vs. respective body weights at day 0; **P<0.05 vs. respective body weight s at days 0, 1, 3, and 7; ^p<0.05 vs. CI+DMEM. CI: radiation + wound; DMEM: Dulbecco’s Modified Eagle’s Medium

It is evident that RI reduced body weight and CI further reduced body weight [5]. Figure 4 shows nadir body weight losses of 14 days and 21 days after 9.25 Gy CI (Figure 4a) and 9.75 Gy CI (Figure 4b), respectively. The surviving CI mice treated with BMSCs were capable of regaining their body weights at days 14 and 21 in CI mice irradiated at 9.25 Gy (Figure 4a), although the body weight data between vehicle- and BMSCs-treated groups show no significant differences in CI mice irradiated at 9.75 Gy, but the BMSCs-treated surviving mice almost recovered their body weights by day 30 (Figure 4b).

### Water consumption

**Figure 5:**
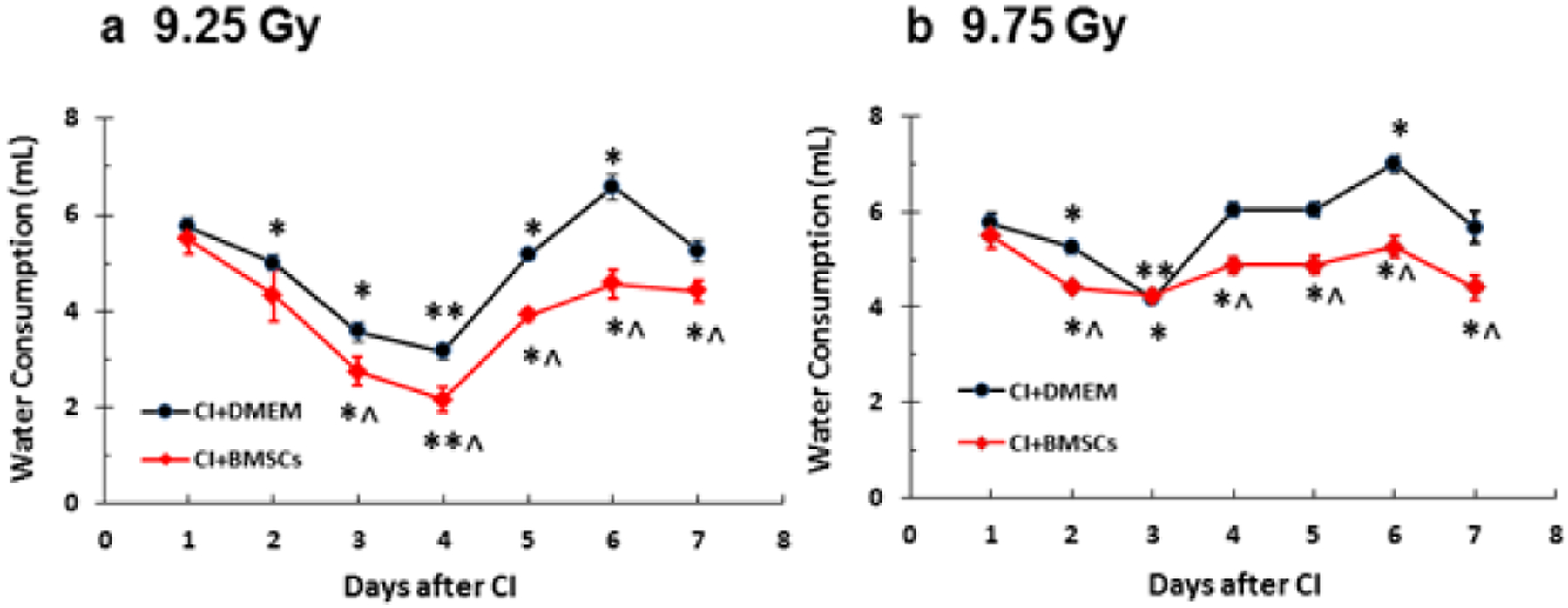
BMSCs reduced water consumption after whole-body ionizing irradiation combined with skin wound (CI). B6D2F1/J mice were exposed to *γ*-radiation followed by 15% TBSA skin-wound trauma. BMSCs (approximately 3 × 106 cells per mouse) were injected via tail vein 24 h after CI. N=10–11 per group and repeated once. a. 9.25 Gy. b. 9.75 Gy. *p<0.05 vs. respective water consumption volumes at day 1; **p<0.05 vs. respective water consumption columns at days 1, 2, and 3; ^p<0.05 vs. CI+DMEM. CI: radiation + wound; DMEM: Dulbecco’s Modified Eagle’s Medium

It is evident that CI stimulates water consumption, but these changes return to the normal level by the 7^th^ day after CI [5]. The BMSC treatment significantly reduced water consumption on days 3–7 in CI mice irradiated at 9.25 Gy (Figure 5a) and on days 2, and 4–7 in CI mice irradiated at 9.75 Gy (Figure 5b).

### Wound healing

**Figure 6:**
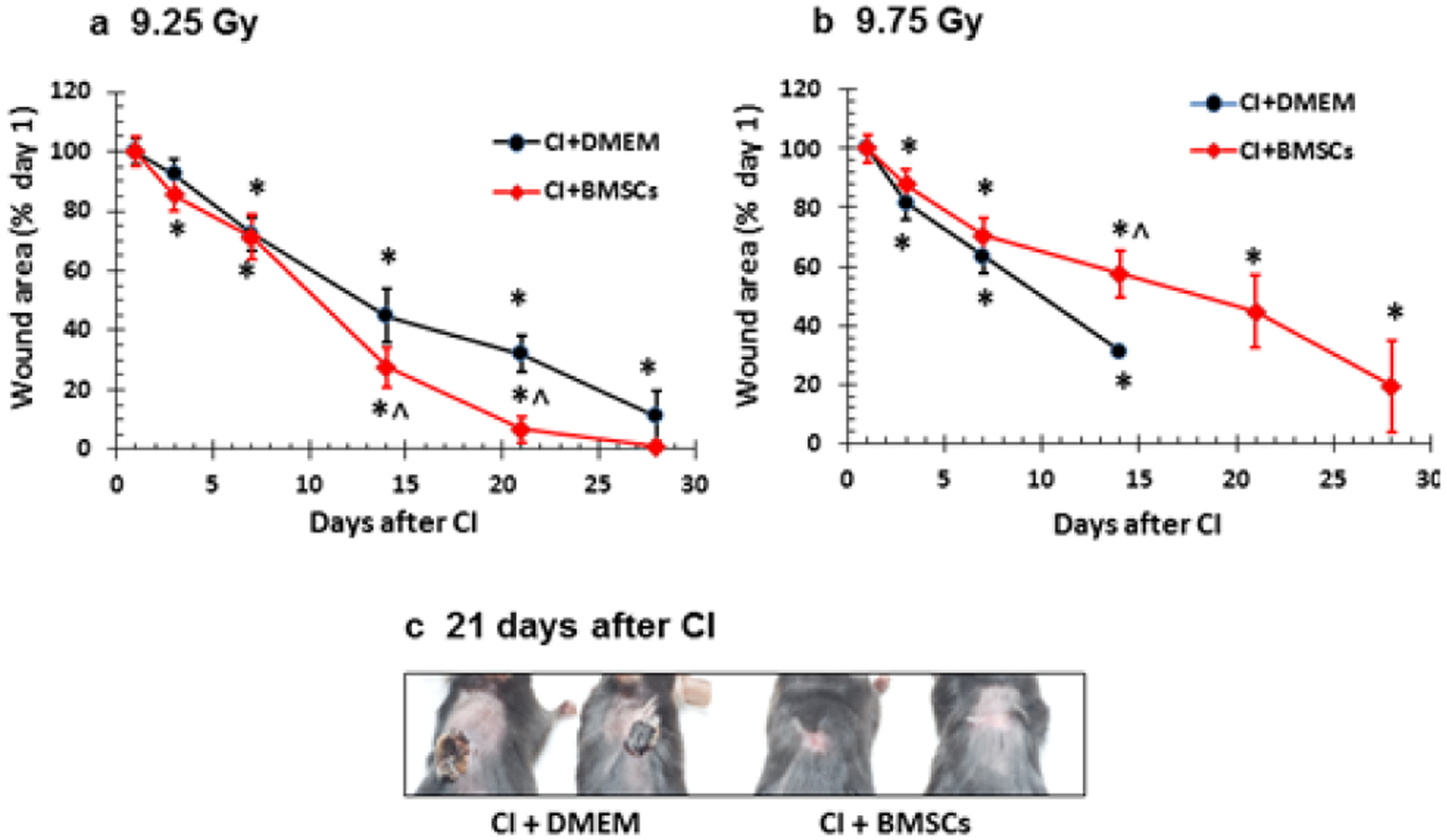
BMSCs accelerated wound healing after whole-body ionizing irradiation combined with skin wound (CI). B6D2F1/J mice were exposed to *γ*-radiation followed by 15% TBSA skin-wound trauma. BMSCs (approximately 3 × 106 cells per mouse) were injected via tail vein 24 h after CI. N=10–11 per group, repeated once. a. 9.25 Gy. Wound was fully closed by day 21 after CI. *P<0.05 vs. CI+DMEM. b. 9.75 Gy. Wound was not fully healed by day 30 after CI. c. Representatives of wound closure on the back of mice. Mice were exposed to 9.25 Gy followed by 15% TBSA skin-wound trauma. BMSCs (approximately 3 × 106 cells per mouse) were injected via tail vein 24 h after CI. *p<0.05 vs. respective wound areas at day 1; ^p<0.05 vs. CI+DMEM. CI: radiation + wound; DMEM: Dulbecco’s Modified Eagle’s Medium

Radiation is known to delay wound healing [4]. In non-irradiated B6D2F1 mice, skin wound normally healed within 14 days, while in irradiated mice, skin wound took more than 30 days to heal [5]. BMSC treatment accelerated the wound healing rate from 5.7 mm^2^/day to 7.6 mm^2^/day in CI mice irradiated at 9.25 Gy (Figure 6a) but not at 9.75 Gy (Figure 6b). However, Figure 6b shows that the BMSCs-treated surviving mice continued to heal the wound by day 30.

Figure 6c shows representative wound healing by 21 days in CI mice irradiated at 9.25 Gy post-treated with BMSCs.

### Bone marrow histopathology

**Figure 7:**
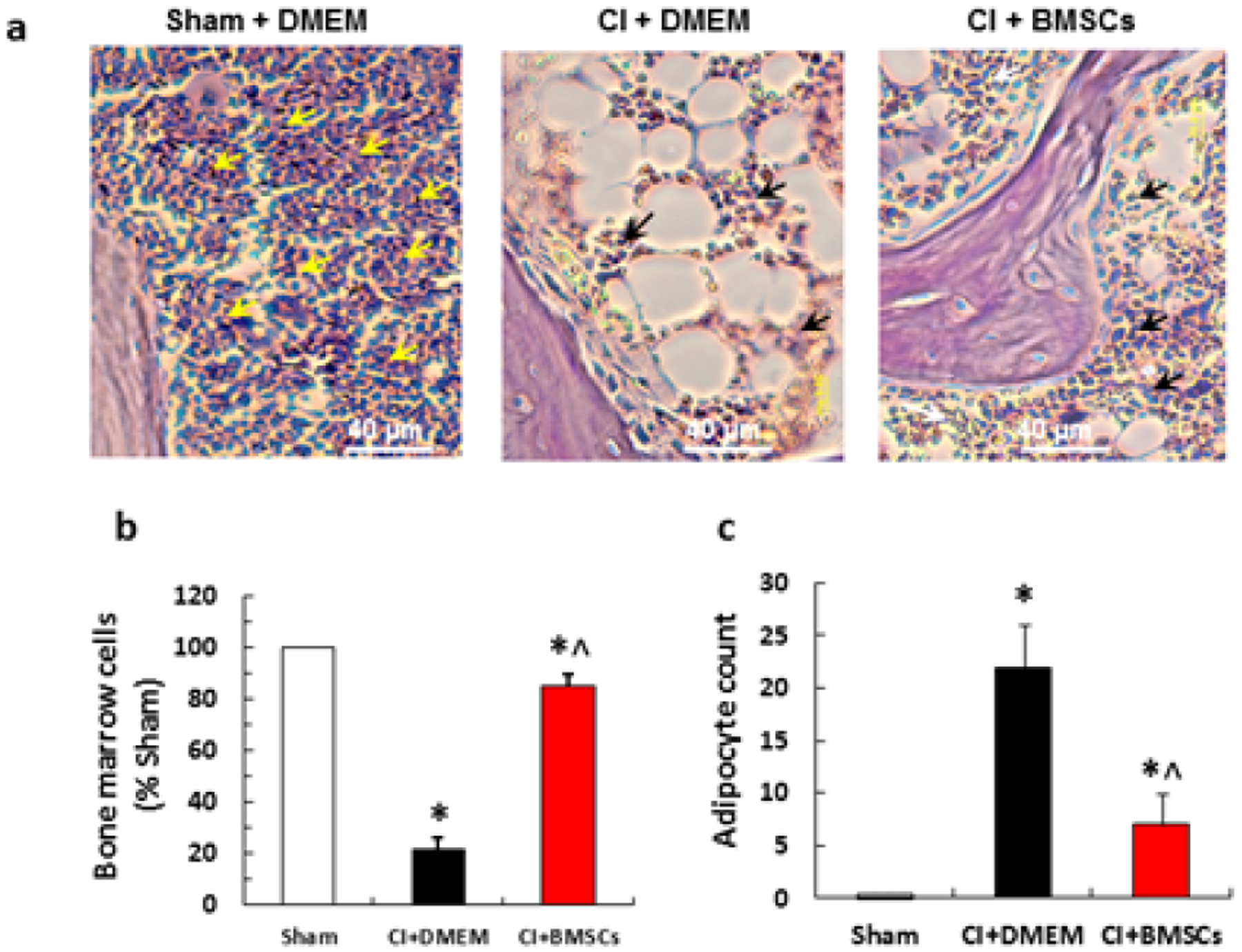
BMSCs increased bone marrow cellularity at day 30 after whole-body ionizing irradiation combined with skin wound (CI). a. Histology of bone marrow specimens (H&E staining). Hematopoietic cells are indicated with either yellow or black arrows. b and c. Relative counts of bone marrow cells. Experimental conditions: Mice were exposed to 9.25 Gy followed by 15% TBSA skin-wound trauma. BMSCs (approximately 3 × 106 cells per mouse) were injected via tail vein 24 h after CI. N=6 per group. CI: combined injury; DMEM: Dulbecco’s Modified Eagle’s Medium *P<0.05 vs. sham; ^p<0.05 vs. CI+DMEM. CI: radiation + wound; DMEM: Dulbecco’s Modified Eagle’s Medium

CI significantly reduced the number of bone marrow cells and increased the number of adipocytes. Treatment with BMSCs increased the bone marrow cells and reduced the number of adipocytes (Figure7).

## Discussion

This report presents novel data that BMSC therapy significantly decreased radiation combined injury-induced mortality, body-weight loss, and water consumption, but accelerated the wound healing rate. The observations were consistent with our previous publications on G-CSF therapy [7], ciprofloxacin therapy [8] or ghrelin therapy [36]. Body weight loss was thought to be due to entheropathy associated with the hematopoietic acute radiation syndrome [4,37]. The increase in water consumption could be a result of dehydration due to (i) fluid release through open wounds, and (ii) systemic metabolic response to the trauma [38,39]. The delayed wound healing partly was due to increased inflammation caused by bacterial infection resulting from the open wound [4] and radiation-injured repairing mechanisms at the skin [4]. Moreover, numerous recent reports indicate that efficient regeneration and recovery from penetrating wounds, contusions and radiation-related injury require recruitment of endothelial, epithelial, myeloid, and mesenchymal lineages of multipotent cells from surrounding environment, the peripheral blood, and bone marrow at site of damage [40,41]. This pathphysiological response can be skewed after total body irradiation producing deleterious effects on cell proliferation and clonogenicity, and, in particular, due to a dramatic cellular depletion in bone marrow, the major source of the essential multipotent cells and leukocytes.

Emerging perspective on physiological role of mesenchymal stromal cells (MSCs) in tissues came with their new applications in cell therapy, regenerative medicine and tissue engineering [25]. With this consideration, we employed Mesenchymal stromal cells originated from bone marrow (BMStroCs) to mitigate CI sickness and improve wound recovery in mice. BMStroCs were identified as colony-forming unit-fibroblasts (CFU-Fs) in 1970 [24] and their tri-lineage potential was described in 1999 [42]. Because BMStroCs possess multipotentiality and relative ease of isolation from many tissues, they have great appeal for tissue engineering and therapeutic applications [43]. Stro-1 is a well-known marker on BMStroCs [44]. Our BMSCs displayed Stro-1, validating the identity of BMStroCs. However, Stro-1 expression could be lost during culture expansion [45]. Therefore, our BMSCs were identified in conjunction with positivity of CD44 and Sca-1 expression [27].

Our laboratory has found that BMSC administration did not increase 30-day survival after radiation in our experiments. The result is in agreement with the data obtained in an experiment with 5-week-old female BALB/c mice given 9 Gy whole-body irradiation 24h prior to i.v. injections of BMSCs [27]. This failure suggests that hematopoietic progenitor stem cells are required. In that publication [27], SOD-transfected BMSCs successfully increased survival, confirming the oxidative free radicles are detrimental. In contrast to RI, we found that BMSC administration significantly improved 30-day survival of CI mice, suggesting that tissue damage caused by CI may contribute to lethality. Bone marrow transplantation enhanced survival after CI [9], inhibited the RI-induced autophagy in crypt cells of the ileum [46], and restored the intestinal mucosal barrier after RI [29]. CI resulted in severe intestinal injury [4]. The possibility of BMSC therapy improving intestinal structure after CI cannot be ruled out.

Our laboratory reported that CI injured mice ileum [4] and reduced body weight [5]. Herein we report that BMSCs attenuated body weight loss after CI. This attenuation could be due to the recovery of intestinal injury induced by BMSCs increasing numbers of Lgr5-positive stem cells in crypts [47,48]. Moreover, BMSCs reversed the suppression of water intake by irradiation, an important index of recovery. It is documented that CI magnifies RI-induced increases in cytokines [4]. Because treatment with BMSCs has been demonstrated to reduce IL-6, IL-10, IL-12A, and IL-17 and elevate G-CSF and GM-CSF [29,47], we surmise that it is highly likely that this capacity also contributes to the survival improvement after CI observed in this report.

Our laboratory has shown that radiation delayed wound healing [4,5]. Treatment with BMSCs effectively shortened the wound-healing duration yet the underlying mechanism is unclear. This result is in agreement with findings on the radiation-induced skin burn healing [30,31]. Because CI reduced cadherin-6 (extracellular adhesive molecule), increased MMPS (inhibitors of extracellular matrix), TLRs, and Myd88 (coupled with TLR-4) of the skin next to the wounded area [49], we postulate that BMSCs may restore cadherin-6 and reduce MMPs in wounded skin. Other signal transduction pathways shown to be involved in CI such as iNOS [4], PI3-K/Akt, ERK1/2, JNK, p38 [50], and p53 [51] cannot be excluded in this BMSC-induced acceleration of wound healing. Our preliminary *in vitro* data suggest that radiation activated AKT and MAPK signaling network in BMSCs (Kiang, unpublished data). More *ex vivo* experiments with each specific inhibitor for each signal molecule to tear out their roles in cell survival are undergoing in our laboratory. Interestingly, *in vitro* challenge of BMSCs with *Staphylococcus epidermidis* can induce the autophagy pathway to execute antibacterial defense response, and that homeostatic shift due to the bacteria-induced stress includes mitochondrial remodeling and sequestration of the compromised organelles via mitophagy [52]. It is not clear whether BMSCs develop abnormal chromosome numbers under this condition, which needs to be further explored.

Treatment of CI mice with BMSCs may be expected to improve bone marrow morphology and, indeed, such was the case. BMSCs significantly restored bone marrow cellularity. BMSCs are sensitive to ionizing radiation. It has been reported that radiation increases activities of NF-kB, SUMO1, LC3, and MMPs [53], a harmful effect on BMSCs. Therefore, it may not be favorable to administer BMSCs prior to irradiation.

In summary, BMSC therapy significantly increased survival, reduced body weight loss, decreased water consumption, and accelerated wound healing. These results suggest that BMSCs are potentially therapeutic for treating CI.

## Conclusions

We demonstrate the treatment potential of BMSCs for wound injuries in mice. BMSC therapy is efficacious to sustain animal survival after CI and accelerate wound healing. Our results, taken together with the results of previous studies by other laboratories [54] as well as several clinical trials using MSCs to treat injuries including spinal injury and myocardial infarction, show that BMSCs facilitate regenerative medicine and will promote cell therapy in future.

## Abbreviations:

AFRRI: Armed Forces Radiobiology Research Institute
BMSC: Bone Marrow Mesenchymal Stem Cells
BMStroCs: Bone Marrow Mesenchymal Stromal Cells
CFU-F: Colony-Forming Units-Fibroblast
CI: Combined Injury
DMEM: Dulbecco’s Modified Eagle’s Medium
DMF: Dose Modifying Factor
i.p.: Intraperitoneal
LC-3: Light Chain 3
MMPs: Matrix Metalloproteinases
MOD: Multi-Organ Dysfunction
MOF: Multi-Organ Failure
Myd88: Myeloid Differentiation Primary Response Gene (88)
NF-kB: Nuclear Factor-KeppaB
*Oct-4*: Octomer-Binding Transcription Factor 4
p.o.: Oral Gavage
RBC: Red Blood Cells
RCI: Radiation Combined Injury
*rex-1*: same as Zfp-42
RI: Radiation Injury
i.v.: Intraveneous
Sca-1: Stem Cell Antigen 1
SOD: Superoxide Dismutase
*sox-2*: Sex Determining Region Y-box 2
SUMO-1: Small Ubiquitin-Related Modifier-1
TBSA: Total Body Surface Area
TLR: Toll-Like Receptor
VSD: Veterinary Science Department
W: Wound
